# Effects of Maximal Fat Oxidation Exercise Training for Body Composition and Cardiovascular Risk Factors on College Students With Obesity

**DOI:** 10.31083/RCM46637

**Published:** 2026-04-17

**Authors:** Jin Yang, Haimei Zhu, Feiyi Sun, Zuowei Pei

**Affiliations:** ^1^Department of Central Laboratory, Central Hospital of Dalian University of Technology, 116033 Dalian, Liaoning, China; ^2^Faculty of Medicine, Dalian University of Technology, 116024 Dalian, Liaoning, China; ^3^Health Medical Department, Central Hospital of Dalian University of Technology, 116033 Dalian, Liaoning, China; ^4^Department of Cardiology, Central Hospital of Dalian University of Technology, 116033 Dalian, Liaoning, China

**Keywords:** aerobic exercise, obesity, cardiovascular risk factors, oxidative stress

## Abstract

**Background:**

Cardiovascular disease (CVD) remains the leading cause of mortality among various diseases in China, with both the incidence and mortality rates associated with CVD continuing to rise. Obesity, as a key risk factor for CVD, exacerbates the disease burden. Concurrently, the rates of overweight and obese individuals among Chinese college students have been increasing annually. Maximal fat oxidation (FATmax)-intensity training, which precisely identifies the optimal exercise intensity for fat oxidation, can effectively improve cardiorespiratory function, regulate metabolic levels, and reduce the risk of chronic diseases. Thus, this study aimed to investigate the effects of FATmax-intensity exercise on cardiovascular disease risk factors in obese college students and to explore the associated underlying mechanisms.

**Methods:**

A longitudinal single-group pre–post experimental design was adopted, with a 12-week intervention conducted on 24 obese college students. Measurements and comparisons of body composition, biochemical indicators, blood parameters, cardiorespiratory function, oxidative stress-related indicators, and immunoinflammatory cytokines were performed before and after the intervention.

**Results:**

The results demonstrated that FATmax-intensity training significantly reduced the body weight, body mass index (BMI), body fat percentage, waist-to-hip ratio, abdominal adipose tissue, subcutaneous fat, resting heart rate, endothelin-1 (ET-1), C–X–C chemokine receptor 1 (CXCR1), CXCR2, granulocyte–macrophage colony-stimulating factor (GM-CSF), interferon-gamma (IFN-γ) and interleukin-33 (IL-33) (*p* < 0.05) values in participants, while significantly increasing peak oxygen uptake (peak VO_2_), anaerobic threshold, the ratio of forced expiratory volume in one second to forced vital capacity (FEV1/FVC), maximal mid-expiratory flow (MMEF), endothelial nitric oxide synthase (eNOS), and vascular endothelial growth factor (VEGF) (*p* < 0.05).

**Conclusions:**

These findings provide preliminary evidence that applying FATmax-intensity exercise improves body composition, oxidative stress indicators, immunomodulatory anti-inflammatory function, and reduces cardiovascular disease risk in young obese populations, thereby providing the foundation for further research on the effects of FATmax-intensity exercise on other cardiovascular risk factors and potential mechanisms.

## 1. Introduction

Cardiovascular diseases (CVD) are the leading cause of death globally and constitute a major public health challenge. They not only compromise individuals’ health and shorten lifespan, but also substantially reduce patients’ quality of life. In addition, they place a significant burden on global healthcare systems due to the high medical costs associated with their management [1]. The CVD burden is particularly severe in China, where it has consistently remained the leading cause of death among both urban and rural residents. By 2023, the number of CVD patients in China was estimated to reach 330 million, with no indication of a turning point in the growing disease burden [2]. Obesity, being one of the key pathogenic risk factors for CVD, can directly drive its onset and progression through metabolic and inflammatory pathways [3]. However, the current global prevalence of obesity is alarming: in 2021, approximately 211 million adults aged 25 and above were overweight or obese, accounting for nearly half of the global adult population [4]. Moreover, the issue of obesity among Chinese college students is becoming increasingly prominent and showing a continuous upward trend [5, 6].

Aerobic training, which focuses on aerobic metabolism, has been widely validated as an effective intervention for enhancing cardiorespiratory function, improving metabolic indicators, and reducing the risk of chronic diseases such as obesity and CVD [7, 8]. It has been established as a crucial measure for the primary prevention of CVD [9]. To maximize the fat-reducing effects of aerobic training, Jeukendrup and Achten [10] introduced the concept of “maximal fat oxidation (FATmax)-intensity” in 2001. This refers to the individualized exercise intensity corresponding to the peak rate of fat oxidation that occurs during a given time period [10]. FATmax training typically involves low to moderate exercise intensity efficiently mobilizing fat for energy, promoting fat reduction while preserving lean body mass, thereby effectively improving body composition, making it particularly suitable for overweight and obese populations [11, 12, 13].

In summary, given the high incidence and growing burden of CVD, the rising prevalence of obesity among college students, and the clear potential of FATmax training in improving cardiovascular health, this study aims to thoroughly investigate the relationship between FATmax-intensity training, obesity, and cardiovascular health outcomes. It will also explore the underlying mechanisms, providing a scientific basis for more precise and effective strategies for the prevention and intervention of CVD.

## 2. Methods

### 2.1 Study Design and Participants

This study implemented a single-group pre-post design without a control group. Participants were recruited from Dalian University of Technology. The inclusion criteria included: primary obesity, body mass index (BMI) ≥28 kg/m^2^, and status as a non-graduating student. Exclusion criteria were: diseases affecting exercise capacity, regular exercise habits within the past six months, diagnosed cardiovascular or respiratory diseases, current use of medications that may affect cardiovascular function, liver function, or metabolic function, long-term alcohol abuse or current smoking, pregnancy or planning of pregnancy, mental or cognitive impairments, and irregular eating patterns or extreme dieting [14].

Initially, 24 participants were recruited, but two withdrew for personal reasons, resulting in a final sample size of 22 (18 males, four females), with an average age of 22.14 ± 2.99 years. The average BMI was 32.28 ± 3.24 kg/m^2^, and the average height was 1.76 ± 0.07 m. All participants provided informed consent.

### 2.2 Exercise Intensity Setting

All participants underwent cardiopulmonary exercise testing (CPET) through an incremental load exercise test. Oxygen consumption (VO_2_) and carbon dioxide output (VCO_2_) were continuously monitored to calculate the fat oxidation rate at each power level using the formula [15]:

Fat oxidation rate (g/min) = 1.6946 × VO_2_ (L/min) – 1.7012 × VCO_2_ (L/min).

The heart rate corresponding to FATmax was set as the target exercise heart rate.

### 2.3 Training Protocol

Starting from March 1, 2025, participants engaged in a 12-week aerobic exercise intervention, with progress tracked via a check-in system. The exercise frequency was 4–5 times per week, with each session lasting 45–60 minutes. Intensity was maintained within the target heart rate range corresponding to maximal fat oxidation. Participants could choose the mode of exercise based on their preference and circumstances, including running, rope skipping, yoga, etc. Additionally, starting from the 4th week of their training program, participants were encouraged to incorporate three sessions of resistance training per week. This was to overcome the weight loss plateaus and accelerate the improvement of cardiovascular risk factors. Each session included a 10-minute warm-up before exercise and a 10-minute cool-down after exercise to prevent injuries.

### 2.4 Main Reagents, Instruments, and Methods

Body composition data, including body fat mass, muscle mass, body fat percentage, and visceral fat area, were assessed using a professional body composition analyzer X‑SCAN PLUS Ⅱ (Gyeongsan-si, Gyeongsangbuk-do, South Korea), manufactured by SELVAS Healthcare from South Korea. All participants emptied their bladders before undergoing body composition analysis. In the early morning, 5 mL of fasting venous blood was collected from each subject and injected into vacuum tubes containing an anticoagulant. Samples were analyzed using an automated biochemical analyzer ADVIA CHEMISTRY XPT, manufactured by SIEMENS from Japan, to obtain the values of biochemical indicators such as blood glucose, blood lipids (total cholesterol, triglycerides, high-density lipoprotein cholesterol, low-density lipoprotein cholesterol), and liver and kidney functions (alanine aminotransferase, aspartate aminotransferase, creatinine, urea nitrogen, etc.). Similarly, 2 mL of fasting venous blood was collected in the early morning in ethylenediaminetetraacetic acid dipotassium salt (EDTA-K2) anticoagulant tubes. Hematological parameters such as white blood cell count, red blood cell count, hemoglobin concentration, and platelet count in the blood samples were measured by the Mindray CAL8000 automatic hematology analysis line, which was manufactured by Mindray in Guangdong, China. The cardiopulmonary exercise testing of the subjects was conducted using the exercise cardiopulmonary tester Master Screen CPX (Hoechberg, Bavaria, Germany), manufactured by Vyaire Medical GmbH from Germany.

Before blood sample collection, participants fasted for at least 12 hours, abstained from caffeine for 12 hours, and avoided alcohol and strenuous exercise for 24 hours. Venous blood (5 mL) was collected in the morning under fasting conditions, centrifuged at 1000 ×g for 20 minutes at room temperature, and serum was separated and stored at –80 ℃ for later analysis. Enzyme-Linked Immunosorbent Assay (ELISA) was employed for the detection and quantification of oxidative stress-related indicators and immunoinflammatory cytokines. The ELISA kits used in the experiment were all purchased from MEIMIAN Industrial Co., Ltd., Jiangsu, China. The microplate reader used was the Infinite 200 PRO (Grödig, Salzburg, Austria), manufactured by TECAN from Austria.

### 2.5 Statistical Method

Data were statistically analyzed using GraphPad Prism 9.0 (GraphPad Software, Inc., San Diego, CA, USA). The Shapiro-Wilk test was used for normality testing. Data are presented as mean ± SD for normally distributed variables or median (Q1, Q3) for non-normally distributed variables. For variables with a normal distribution or whose differences before and after intervention conformed to a normal distribution, a paired *t*-test was used to compare the differences before and after the intervention. For variables with a non-normal distribution and whose differences before and after intervention also failed to conform to a normal distribution, the Wilcoxon rank-sum test was applied to analyze the differences between pre-intervention and post-intervention, with *p* < 0.05 considered statistically significant.

## 3. Results

### 3.1 Effects of 12-Week Exercise Training on Body Composition in College Students

After completing the training, the following significant changes in the body weight composition were observed compared to before training: Significant reductions were observed in body weight (*p* < 0.001), BMI (*p* < 0.001), body fat percentage (*p* < 0.01),the waist-to-hip ratio (WHR) (*p* < 0.01), visceral fat (*p* < 0.001), and subcutaneous fat (*p* < 0.001). Additionally, significant reductions were observed in body water (*p* < 0.01) and intracellular water (*p* < 0.01). Furthermore, a significant decrease was observed in basic metabolism (*p* < 0.01), whereas no significant changes were detected in muscle mass. The detailed results of the participants’ body composition are shown in Table 1.

**Table 1.** **The body composition of participants**.

	Pre	Post	*p*
Weight (kg)	100.5 ± 13.54	93.91 ± 14.58	<0.001***
BMI (kg/m^2^)	30.93 (29.97, 33.65)	29.37 (27.51, 31.04)	<0.001***
Body fat percentage%^1^	30.5 (28.2, 33.0)	28.7 (26.5, 32.3)	0.003**
Muscle mass^1^ (kg)	25.63 ± 4.45	23.09 ± 4.73	0.091
WHR^1^	0.88 (0.84, 0.89)	0.85 (0.83, 0.88)	0.001**
Visceral fat^1^ (kg)	4.6 (3.6, 5.2)	3.5 (3.2, 4.5)	<0.001***
Subcutaneous fat^1^ (kg)	25.63 ± 4.45	23.09 ± 4.73	<0.001***
Basic metabolism^1^ (kcal)	1820 ± 179.9	1786 ± 190.2	0.003**
Body water^1^ (kg)	49 ± 6.09	47.84 ± 6.44	0.003**
Intracellular water^1^ (kg)	30.8 (28.3, 33.4)	30.6 (26.4, 32.8)	0.001**
Extracellular water^1^ (kg)	18.62 ± 2.81	18.51 ± 2.49	0.649

Abbreviation: BMI, body mass index; WHR, waist-to-hip ratio. Data are means ± SD or median (Q1, Q3). Significantly different from Pre-training: **p < 0.01, ***p < 0.001. Overall N = 22; ^1^N = 19 for indicated variables (3 participants missed the post-intervention body composition analysis due to personal reasons).

### 3.2 Effects of 12-Week FATmax-Intensity Exercise Training on Biochemical Indicators in College Students

After the training period, significant reductions were observed in alanine aminotransferase (ALT) (*p* < 0.05), and aspartate aminotransferase (AST) (*p* < 0.05). However, the other biochemical indicators, such as high-density lipoprotein cholesterol (HDL-C) and low-density lipoprotein cholesterol (LDL-C) did not show significant changes. The results of the participants’ biochemical indicators are summarized in Table 2.

**Table 2.** **The biochemical indicators of the participants**.

	Pre	Post	*p*
ALT (U/L)	36.5 (17.5, 62.5)	26.0 (14.75, 36.0)	0.008**
AST (U/L)	24.5 (17.0, 39.25)	19.0 (17.0, 22.0)	0.007**
TP (g/L)	76.65 (74.30, 79.48)	75.6 (74.2, 79.0)	0.753
ALB (g/L)	48.2 ± 2.22	47.74 ± 2.58	0.451
GLB (g/L)	28.62 ± 2.599	28.7 ± 1.93	0.636
A/G	1.7 ± 0.19	1.65 ± 0.17	0.143
GGT (U/L)	32.5 (19.0, 48.0)	28.0 (16.5, 41.0)	0.253
ALP (U/L)	81.0 (68.5, 104.8)	80.5 (53.5, 91.5)	0.007**
TBIL (µmol/L)	15.4 (11.5, 21.05)	12.55 (11.38, 16.93)	0.086
DBIL (µmol/L)	5.9 (3.95, 7.17)	4.5 (3.97, 6.0)	0.112
IBIL (µmol/L)	10.22 ± 4.08	9.10 ± 3.66	0.196
GLU (mmol/L)	4.59 ± 0.47	4.66 ± 0.32	0.420
UREA (mmol/L)	4.73 (4.18, 5.18)	4.40 (3.81, 5.27)	0.301
CREA (µmol/L)	69.5 (62.78, 78.65)	72.8 (64.95, 81.05)	0.419
eGFR-Cr [mL/(min·1.73 m^2^)]	130.9 (120.3, 143.7)	131.7 (114.9, 140.2)	0.972
UA (µmol/L)	418 ± 93.26	434.8 ± 97.26	0.495
T-CHOL (mmol/L)	4.63 ± 0.36	4.53 ± 0.36	0.265
TG (mmol/L)	1.25 (0.94, 1.47)	1.39 (0.94, 1.77)	0.175
HDL-C (mmol/L)	1.01 (0.91, 1.24)	0.96 (0.87, 1.14)	0.061
LDL-C (mmol/L)	2.70 ± 0.42	2.70 ± 0.41	0.977

Abbreviation: ALT, alanine aminotransferase; AST, aspartate aminotransferase; TP, total protein; ALB, albumin; GLB, globulin; A/G, albumin/globulin ratio; GGT, gamma-glutamyl transferase; ALP, alkaline phosphatase; TBIL, total bilirubin; DBIL, direct bilirubin; IBIL, indirect bilirubin; GLU, glucose; UREA, Urea; CREA, creatinine; eGFR-Cr, estimated Glomerular Filtration Rate (by Creatinine); UA, uric acid; T-CHOL, total cholesterol; TG, triglycerides; HDL-C, high-density lipoprotein cholesterol; LDL-C, low-density lipoprotein cholesterol. N = 22; Data are means ± SD or median (Q1, Q3). Significantly different from Pre-training, **p < 0.01.

### 3.3 Effects of 12-Week FATmax-Intensity Exercise Training on Blood Composition in College Students

Following the training, significant reductions were identified in red blood cell (RBC) (*p* < 0.05), hemoglobin (HGB) (*p* < 0.01), and hematocrit (HCT) (*p* < 0.05). In addition, the neutrophil-to-lymphocyte ratio (NLR) showed no significant changes. The outcomes for the participants’ blood composition are detailed in Table 3.

**Table 3.** **The blood composition of the participants**.

	Pre	Post	*p*
WBC (×10^9^/L)	7.3 (6.78, 8.34)	7.22 (6.72, 8.47)	0.879
Neu%	55.2 ± 7.53	55.5 ± 8.68	0.855
LY%	36.22 ± 6.50	35.72 ± 8.47	0.746
NLR	1.68 (1.2, 1.86)	1.52 (1.32, 1.97)	0.848
MO%	5.8 (5.07, 6.55)	6.2 (5.75, 6.55)	0.238
EO%	1.85 (1.35, 2.67)	1.85 (1.07, 2.6)	0.629
BASO%	0.3 (0.1, 0.42)	0.3 (0.2, 0.45)	0.633
Neu (×10^9^/L)	4.29 (3.28, 4.94)	3.91 (3.58, 4.93)	0.744
LY (×10^9^/L)	2.62 (2.36, 3.05)	2.47 (2.33, 2.79)	0.913
MO (×10^9^/L)	0.42 (0.37, 0.49)	0.46 (0.39, 0.51)	0.351
EO (×10^9^/L)	0.15 (0.08, 0.21)	0.13 (0.08, 0.22)	0.850
BASO (×10^9^/L)	0.02 (0.01, 0.03)	0.02 (0.01, 0.03)	0.306
RBC (×10^12^/L)	5.29 ± 0.31	5.19 ± 0.34	0.029*
HGB (g/L)	155.9 ± 10.23	152.2 ± 9.89	0.006**
HCT (%)	46.78 ± 2.82	45.71 ± 2.62	0.012*
MCV (fL)	88.36 ± 2.54	88.05 ± 2.41	0.185
MCH (pg)	29.43 ± 0.88	29.33 ± 1.04	0.249
MCHC (g/L)	333.3 ± 5.38	332.8 ± 5.69	0.677
RDW-CV (%)	12.99 ± 0.53	12.85 ± 0.60	0.146
RDW-SD (fL)	41.94 ± 1.73	41.31 ± 1.95	0.615
PLT (×10^9^/L)	272.5 (243.8, 294.3)	254.5 (243, 285.3)	0.192
PCT (%)	0.27 ± 0.04	0.26 ± 0.04	0.134
MPV (fL)	9.91 ± 0.60	9.88 ± 0.68	0.737
PDW (%)	16.05 (15.88, 16.3)	16 (15.9, 16.23)	0.818

Abbreviation: WBC, white blood cell; NEU, neutrophil; LY, lymphocyte; NLR, neutrophil-to-lymphocyte ratio; MO, monocyte; EO, eosinophil; BASO, basophil; RBC, red blood cell; HGB, hemoglobin; HCT, hematocrit; MCV, mean corpuscular volume; MCH, mean corpuscular hemoglobin; MCHC, mean corpuscular hemoglobin concentration; RDW-CV, red cell distribution width-coefficient of variation; RDW-SD, red cell distribution width-standard deviation; PLT, platelet; PCT, plateletcrit; MPV, mean platelet volume; PDW, platelet distribution width. N = 22; Data are means ± SD or median (Q1, Q3). Significantly different from Pre-training, *p < 0.05, **p < 0.01.

### 3.4 Effects of 12-Week FATmax-Intensity Exercise Training on Cardiorespiratory Function in College Students

The training resulted in significant improvements in cardiorespiratory function. Peak VO_2_ (*p* < 0.001), anaerobic threshold (AT) (*p* < 0.01), the percentage of predicted peak VO_2_ (*p* < 0.01), FEV1/FVC ratio (*p* < 0.01), and the maximal mid-expiratory flow (MMEF) (*p* < 0.05) showed a significant increase after the training. Conversely, significant reductions were identified in resting diastolic blood pressure (DBP) (*p* < 0.05) and resting heart rate (HR) (*p* < 0.05). Furthermore, no significant changes were observed in minute ventilation/carbon dioxide output (VE/VCO2) slope and maximum voluntary ventilation (MVV). The results of the participants’ cardiac function and pulmonary ventilation function during exercise are shown in Table 4.

**Table 4.** **The indicators of the participants’ cardiac function and pulmonary ventilation function during exercise**.

	Pre	Post	*p*
Peak VO_2_ (mL/min)	26.63 ± 4.05	31.49 ± 5.77	<0.001***
AT (mL/min)	17.35 ± 2.37	20.37 ± 4.02	0.003**
Peak VO_2_/pred%	79.27 ± 9.95	88.95 ± 11.77	0.003**
AT/Peak VO_2_%	66.13 ± 11.23	65.4 ± 10.04	0.781
VE/VCO_2_ slope (L/L)	26.34 ± 6.03	27.05 ± 5.97	0.594
Resting SBP (mmHg)	132.5 (112, 138)	120 (106.5, 129.8)	0.068
Resting DBP (mmHg)	81.4 ± 15.09	72.05 ± 13.08	0.040*
SBP max (mmHg)	181.2 ± 30.45	184.1 ± 25.06	0.672
DBP max (mmHg)	73.2 ± 15.78	68.05 ± 13.53	0.244
Resting HR (1/min)	96.25 ± 12.51	88.4 ± 13.46	0.039*
AT HR (1/min)	136.6 ± 13.28	144.3 ± 16.7	0.086
HRmax (1/min)	191.4 ± 16.63	196.2 ± 12.34	0.344
HRmax/pred%	99.74 (92.74, 102.5)	97.97 (96.87, 101)	0.430
FEV1/FVC%	73.11 ± 6.34	78.66 ± 7.03	0.004**
MMEF (L/s)	3.31 ± 0.79	3.75 ± 0.85	0.022*
PEF (L/s)	7.05 ± 1.65	7.60 ± 1.80	0.189
MVV (L/min)	140.6 ± 28.05	137.1 ± 23.55	0.458

Abbreviation: Peak VO_2_, peak oxygen uptake; AT, anaerobic threshold; HR, heart rate; VE, minute ventilation; VCO_2_, carbon dioxide output; SBP, systolic blood pressure; DBP, diastolic blood pressure; FEV1, forced expiratory volume in one second; FVC, forced vital capacity; MMEF, maximal mid-expiratory flow; PEF, peak expiratory flow; MVV, maximum voluntary ventilation. Data are means ± SD or median (Q1, Q3). Significantly different from Pre-training, *p < 0.05, **p < 0.01, ***p < 0.001. N = 20 (2 participants missed the post-intervention cardiopulmonary exercise testing due to personal reasons).

### 3.5 Effects of 12-Week FATmax-Intensity Exercise Training on Oxidative Stress in College Students

**Fig. 1.**
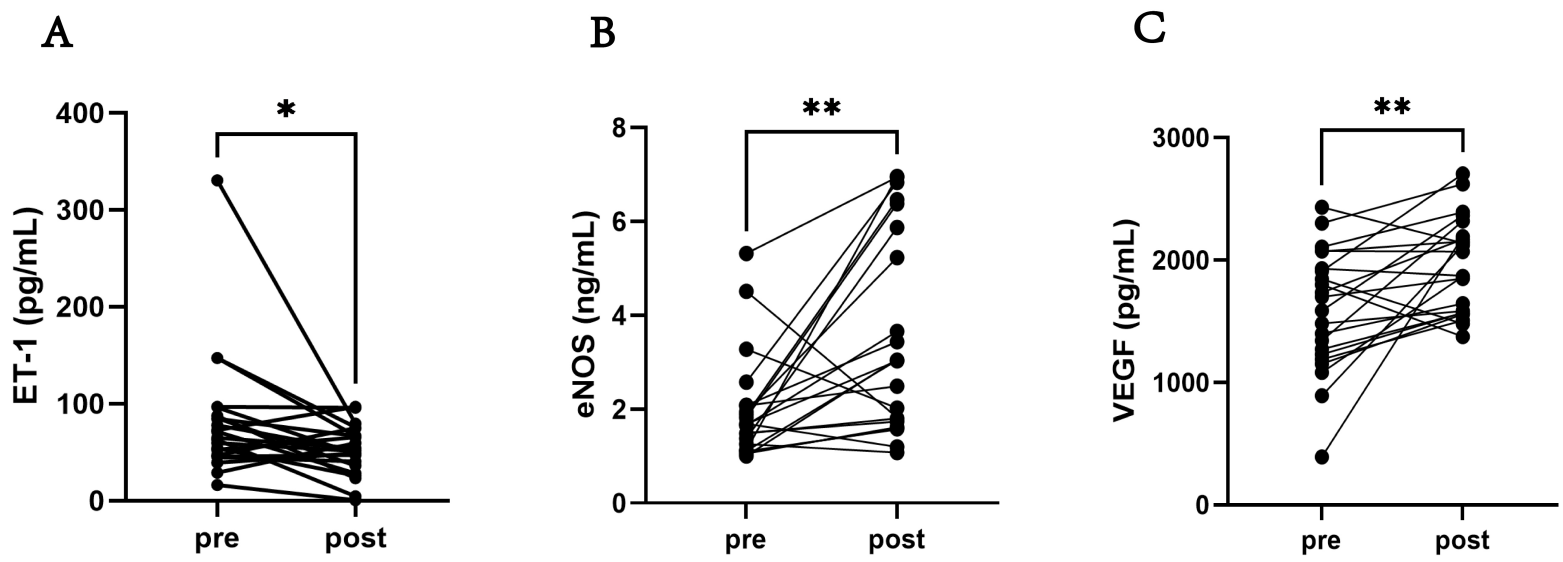
**The oxidative stress-related indicators of the participants**. (A) Comparison of the ET-1 level pre-training and post-training. (B) Comparison of the eNOS level pre-training and post-training. (C) Comparison of VEGF level pre-training and post-training. Abbreviation: ET-1, endothelin-1; eNOS, endothelial nitric oxide synthase; VEGF, vascular endothelial growth factor. N = 22; Data are expressed as means ± SD or median (Q1, Q3). Significantly different from pre-training, *p < 0.05, **p < 0.01.

After training, the endothelin-1 (ET-1) level was significantly decreased (*p* < 0.05). Meanwhile, levels of eNOS (*p* < 0.05), and vascular endothelial growth factor (VEGF) (*p* < 0.05) showed a significant increase after the training. The results of the participants’ oxidative stress markers are shown in Fig. 1.

### 3.6 Effects of 12-Week FATmax-Intensity Exercise Training on Immunomodulatory Anti-Inflammatory Function in College Students

**Fig. 2.**
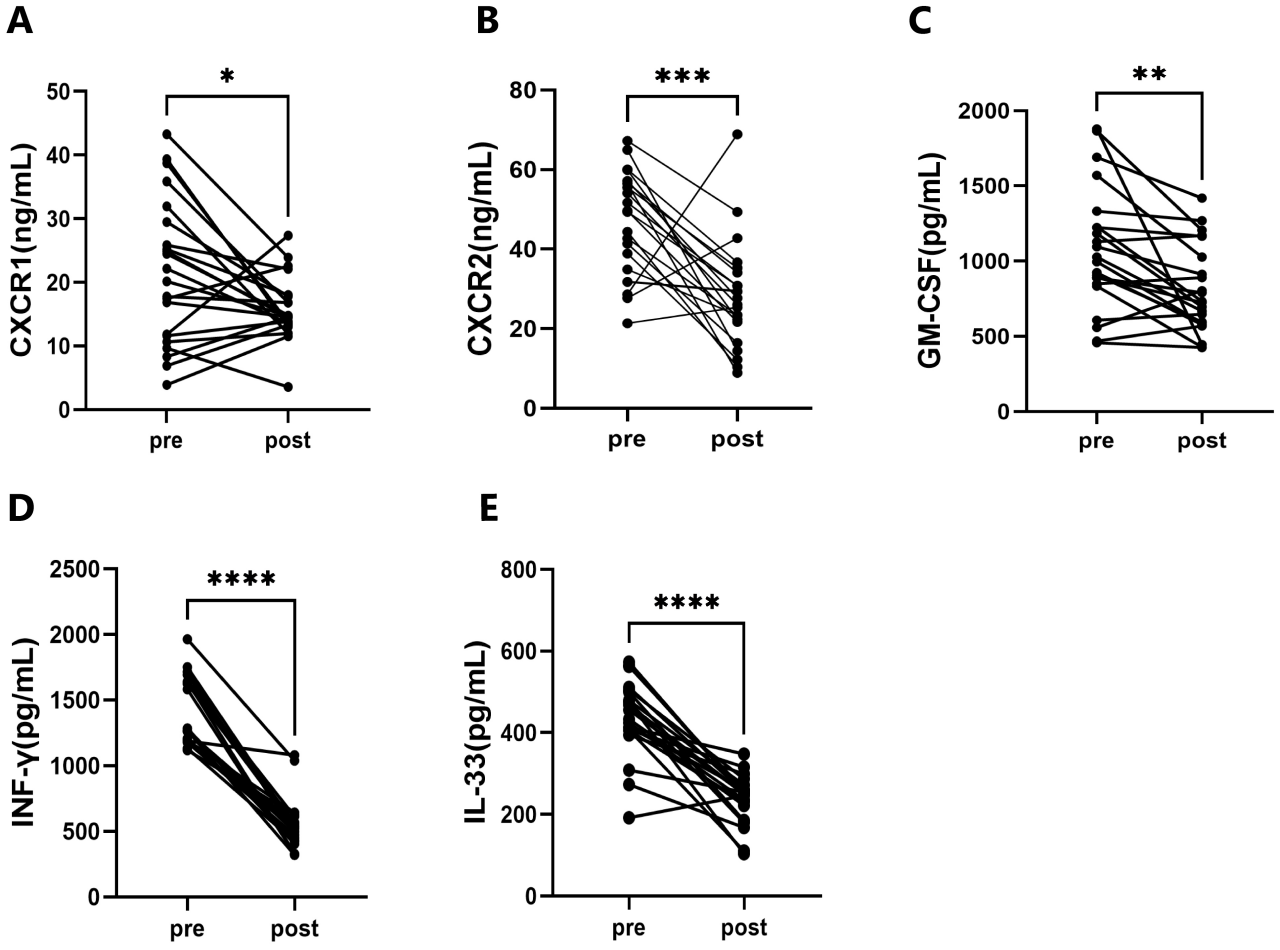
**The Immunoinflammatory cytokines of the participants**. (A) Comparison of CXCR1 pre- and post-training. (B) Comparison of CXCR2 pre- and post-training. (C) Comparison of GM-CSF pre- and post-training. (D) Comparison of IFN-γ pre- and post-training. (E) Comparison of IL-33 pre- and post-training. Abbreviation: CXCR1, C–X–C chemokine receptor 1; CXCR2, C–X–C chemokine receptor 2; GM-CSF, granulocyte–macrophage colony-stimulating factor; IFN-γ, interferon-gamma; IL-33, interleukin-33. N = 22; Data are expressed as means ± SD or median (Q1, Q3). Significantly different from pre-training, *p < 0.05, **p < 0.01, ***p < 0.001, ****p < 0.0001.

After the training program, significant decreases were observed in the levels of C–X–C chemokine receptor 1 (CXCR1) (*p* < 0.05); C–X–C Chemokine Receptor 2 (CXCR2) (*p* < 0.05); granulocyte–macrophage colony-stimulating factor (GM-CSF) (*p* < 0.05); interferon-gamma (IFN-γ) (*p* < 0.05); and interleukin-33 (IL-33) (*p* < 0.05). The results of the participants’ Immunoinflammatory cytokines are shown in Fig. 2.

## 4. Discussion

This study presents evidence that a 12-week FATmax-intensity exercise training intervention can improve body composition, enhance cardiorespiratory function, and reduce CVD risk in college students. The significant improvements observed in various indicators suggest that this training model has potential protective effects on cardiovascular health within the population.

Notably, the study documented significant reductions in body weight, fat mass, and WHR (all *p* < 0.05), indicating effective improvements in disordered lipid metabolism, a crucial factor in lowering the risk of CVD onset [16, 17]. An elevated WHR signifies excessive abdominal fat accumulation, which possesses active endocrine functions and secretes pro-inflammatory cytokines and other harmful hormones [18]. These bioactive substances are known to induce vascular endothelial dysfunction and increase inflammatory responses, which in turn promote the development of atherosclerosis and rising CVD risk. Furthermore, a high WHR is often closely associated with metabolic syndrome, a condition that also contributes to an increased risk of CVD [19]. The reduction in body water and intracellular water likely relates to exercise-induced glycogen depletion, adaptive changes in fluid-regulating hormones, and enhanced protein catabolism due to negative energy balance [20, 21]. It is worth noting that the unexpected decreases in muscle mass and basal metabolic rate may be related to uncontrolled protein intake in the study design, sustained negative energy balance, and a single training modality that lacked resistance training components [22, 23]. Future research studies combining FATmax training with resistance training may better preserve lean body mass while reducing fat, thereby further enhancing metabolic health benefits and reducing CVD risk.

In terms of liver function, the significant reduction in AST activity compared to pre-intervention (*p* < 0.05) suggests improved liver health, which may indirectly reflect enhancements in cardiovascular risk factors [24]. However, no significant changes were observed in HDL-C or LDL-C levels. The lack of change may stem from insufficient exercise stimulus intensity at this specific intensity and modality, along with confounding factors such as the fatty acid composition of the diet, which was not strictly controlled [24, 25, 26, 27]. This limitation is particularly relevant in the context of Chinese university students, who often rely on canteen-prepared meals or takeout food, making it difficult to regulate oil and salt intake and thereby limiting the feasibility of personalized dietary management.

Regarding blood parameters, the observed reduction in red blood cell-related metrics may indicate exercise-induced physiological expansion of blood volume, release of erythropoietin (EPO), and enhanced antioxidant capacity, which are typically markers of good adaptation to regular exercise [28]. FATmax training stimulates the kidneys to release EPO through hypoxic stress; however, the activation of bone marrow hematopoiesis may take two or four weeks. Obese college students may experience more pronounced tissue hypoxia during training due to relatively poor cardiopulmonary function, resulting in a higher peak EPO release. Nevertheless, the rate of erythropoiesis still lags behind the rate of plasma volume expansion, leading to a short-term decrease in RBC, HGB, and HCT. This plasma volume expansion reduces blood viscosity, which can alleviate the low oxygen transport efficiency caused by dyslipidemia and blood hyperviscosity in obese populations, thereby enhancing the fat reduction effect of FATmax training [29]. Meanwhile, a lower HCT can decrease cardiac pumping resistance and reduce cardiovascular pressure in obese individuals with comorbidities such as hypertension and fatty liver disease [30]. It should be noted that if HGB levels drop below 130 g/L in males or 120 g/L in females and persist for more than 4 weeks, symptoms such as decreased maximal oxygen uptake, impaired exercise endurance, and increased fatigue may occur. Additionally, serum ferritin levels below 30 µg/L (or 12 µg/L in specific subgroups) can lead to reduced exercise performance, even if the criteria for overt anemia are not met [31]. The lack of expected change in the NLR may be due to FATmax training being of low to moderate intensity, which is less likely to cause severe inflammation or stress responses [32]. Other factors such as intervention duration, diet, or psychological stress may also have an impact [33]. Future studies could investigate incorporating high-intensity interval training (HIIT) to assess its additional effects on inflammatory markers and CVD risk.

Overall, improvements in cardiorespiratory function were a prominent finding of this study. The significant increases in peak VO_2_, AT, percentage of predicted peak VO_2_, FEV1/FVC ratio, and MMEF (all *p* < 0.05) clearly indicate enhanced cardiorespiratory fitness and respiratory efficiency. The significant reductions in resting HR and DBP (*p* < 0.05) indicate improved cardiac autonomic regulation and a decreased risk of cardiovascular issues [34, 35]. At the same time, there was a slight increase in the anaerobic threshold heart rate and maximum heart rate, indicating an elevation in the participants’ heart rate reserve. This further demonstrates that FATmax exercise enhances cardiac work efficiency and improves cardiovascular health [36]. Long-term regular endurance exercise can enhance respiratory muscle endurance through mechanisms like muscle fiber type transformation and increased mitochondrial content; however, improving respiratory muscle strength requires specialized respiratory muscle training. Obese college students typically have a relatively higher baseline respiratory load, and the proportion of oxygen consumption by respiratory muscles during exercise may be higher. If only FATmax training is performed without targeted respiratory muscle training, there may be little improvement in respiratory muscle strength. This could result in insufficient reduction of dead space ventilation or inadequate improvement in the tidal volume/respiratory rate ratio, ultimately having a limited impact on the VE/VCO_2_ slope [37]. Furthermore, anxiety can stimulate sympathetic nerve excitation, increasing respiratory rate, decreasing tidal volume, and elevating dead space ventilation, which theoretically raises the VE/VCO_2_ slope. If anxiety levels are not controlled or measured in a study, their influence on VE/VCO_2_ may be confounded by “test-day status” [38]. Additionally, cardiopulmonary exercise testing (CPET) requires a stable rhythm and respiratory depth. If participants do not maintain regular breathing as instructed and experience respiratory rhythm fluctuations, the variability of VE/VCO_2_ data will increase, obscuring the true training effect [39]. The unchanged MVV indicates that the training mode may not provide sufficient stimulation for respiratory muscle strength, suggesting that future studies could incorporate respiratory muscle training [40].

A significant decrease in ET-1 indicates that training may have improved vascular endothelial function, reduced vascular contractility, helped lower blood pressure, and alleviated strain on vascular walls, thereby exerting a protective effect on the cardiovascular system [41]. An increase in eNOS enhances vascular diastolic function, reduces peripheral vascular resistance, and promotes blood circulation, which exerts a positive impact on cardiovascular health. An increase in VEGF may help enhance myocardial blood and oxygen supply, promote the repair and regeneration of damaged blood vessels, and mitigate the development and progression of CVDs. Furthermore, VEGF may also play a role in regulating lipid metabolism and energy balance, thereby exerting a further beneficial effect on cardiovascular health [42, 43]. The improvements in these three indicators suggest that FATmax training can reduce the risk of cardiovascular diseases in obese college students through multiple pathways, such as improving vascular endothelial function, regulating vascular vasomotor status, and promoting angiogenesis.

Research has shown that CXCR1 and CXCR2 are closely involved in mediating inflammatory infiltration in cardiovascular diseases by participating in the recruitment and activation of leukocytes. In conditions such as atherosclerosis, CXCR1/2 inhibitors have demonstrated benefits in animal models, including reduced plaque area, improved lipid profiles, relief from ischemia-reperfusion injury, regulation of blood pressure, and limiting cardiac remodeling [44, 45]. GM-CSF can stimulate the proliferation, differentiation, and activation of granulocytes and macrophages, promote the generation of inflammatory cells, and exacerbate inflammatory responses in cardiovascular diseases. The serum GM-CSF level in patients with acute myocardial infarction is significantly correlated with the severity of the disease [46, 47]. IFN-γ regulates the proliferation and migration of vascular smooth muscle cells, influencing vascular remodeling and impairing vascular endothelial function by promoting the expression of inflammatory factors [48, 49]. As a cytokine related to inflammation and immune regulation, IL-33 is associated with obesity-related chronic inflammation. An imbalance of the IL-33/suppression of tumorigenicity 2 (ST2) signaling pathway exacerbates inflammation and leads to myocardial damage [50, 51]. FATmax training induces vascular shear stress, activating eNOS, which promotes the synthesis and release of endothelial nitric oxide (eNO). eNO plays a dual role: it reduces the activity of CXCR1 and CXCR2 on the neutrophils and inhibits the expression of vascular endothelial adhesion molecules, thereby diminishing neutrophil chemotaxis and inflammatory infiltration. Additionally, eNO inhibits the activation of the Nuclear factor kappa-light-chain-enhancer of activated B cells (IFN-κB) pathway, decreasing the synthesis and release of GM-CSF and IFN-γ by macrophages, endothelial cells, T cells, and other cell types. Meanwhile, eNO promotes the degradation of IL-33 protein by regulating proteasome activity and indirectly inhibits IL-33 production through decreasing IFN-γ levels, ultimately achieving a multi-target anti-inflammatory effect [52]. FATmax training can effectively reduce fat accumulation. In the obese state, adipose tissue releases numerous pro-inflammatory factors, and the secretion of these factors declines as adipose tissue is reduced [53]. During exercise, muscles secrete various myokines that exert immunomodulatory effects. These myokines can promote the expansion of regulatory T (Treg) cells, thereby inhibiting the production of IFN-γ and alleviating inflammatory responses [54]. After 12 weeks of FATmax exercise intervention, the serum levels of CXCR1, CXCR2, GM-CSF, IFN-γ, and IL-33 in obese college students decreased significantly, which suggests that exercise may reduce the risk of cardiovascular diseases by inhibiting the chemotaxis, generation, and activation of inflammatory cells, as well as improving vascular endothelial function and the cardiovascular microenvironment.

However, none of the college students participating in the training adopted the recommended intervention of “incorporating resistance training after four weeks of training”. The main reasons included loss of training confidence, lack of suitable training facilities, and scheduling conflicts. In addition, participants did not adhere to the study’s control requirements. To address these challenges, future studies will refine the experimental design to minimize these limitations and develop more comprehensive intervention strategies to further strengthen the training program.

### Limitations

This study has several limitations: short intervention period, limited sample size, the absence of a parallel control group, gender ratio imbalance, and different exercise modes in comparison groups. Future research should expand the sample size, extend the intervention period, establish control groups (e.g., other intensity aerobic exercise groups), and incorporate more comprehensive cardiovascular risk biomarkers. This will help further elucidate the exact effects and mechanisms of FATmax exercise on CVD risk in college students, providing a stronger theoretical basis for its promotion in public health.

## 5. Conclusions

A 12-week FATmax exercise training program can significantly improve college students’ body composition, cardiovascular function, pulmonary function, and oxidative stress markers. It may serve as an effective intervention strategy to reduce the risk of CVD in the college student population. Moreover, combining FATmax training with resistance training and supplementing it with proper dietary management can improve body composition and relevant functional indicators, further reducing CVD risk factors.

## Availability of Data and Materials

The data that support the findings of this study are available from the corresponding author upon reasonable request.
